# Probing the Origins of 1,800 MHz Radio Frequency Electromagnetic Radiation Induced Damage in Mouse Immortalized Germ Cells and Spermatozoa *in vitro*

**DOI:** 10.3389/fpubh.2018.00270

**Published:** 2018-09-21

**Authors:** Brendan J. Houston, Brett Nixon, Bruce V. King, R. John Aitken, Geoffry N. De Iuliis

**Affiliations:** ^1^Priority Research Centre for Reproductive Biology, School of Environmental and Life Sciences, Discipline of Biological Sciences, University of Newcastle, Callaghan, NSW, Australia; ^2^School of Mathematical and Physical Sciences, University of Newcastle, Callaghan, NSW, Australia

**Keywords:** RF-EMR, spermatozoa, oxidative damage, germ cells, DNA damage, mitochondria, mobile phone radiation

## Abstract

As the use of mobile phone devices is now highly prevalent, many studies have sought to evaluate the effects of the radiofrequency-electromagnetic radiation (RF-EMR) on both human health and biology. While several such studies have shown RF-EMR is capable of inducing cellular stress, the physicobiological origin of this stress remains largely unresolved. To explore the effect of RF-EMR on the male reproductive system, we exposed cultured mouse spermatogonial GC1 and spermatocyte GC2 cell lines, as well as cauda epididymal spermatozoa to a waveguide generating continuous wave RF-EMR (1.8 GHz, 0.15 and 1.5 W/kg). This study demonstrated that a 4 h exposure is capable of inducing the generation of mitochondrial reactive oxygen species (ROS) in populations of GC1 (7 vs. 18%; *p* < 0.001) and GC2 cells (11.5 vs. 16 %; *p* < 0.01), identifying Complex III of the electron transport chain (ETC) as the potential source of electrons producing ROS. Assessing the generation of ROS in the presence of an antioxidant, penicillamine, as well as measuring lipid peroxidation via 4-hydroxynonenal levels, indicated that the elevated incidence of ROS generation observed under our exposure conditions did not necessarily induce an overt cellular oxidative stress response. However, exposure to RF-EMR at 0.15 W/kg for 3 h did induce significant DNA fragmentation in spermatozoa (that was no longer significant after 4 h), assessed by the alkaline comet assay (*p* < 0.05). Furthermore, this fragmentation was accompanied by an induction of oxidative DNA damage in the form of 8-hydroxy-2′-deoxyguanosine, which was significant (*p* < 0.05) after spermatozoa were exposed to RF-EMR for 4 h. At this exposure time point, a decline in sperm motility (*p* < 0.05) was also observed. This study contributes new evidence toward elucidating a mechanism to account for the effects of RF-EMR on biological systems, proposing Complex III of the mitochondrial ETC as the key target of this radiation.

## Introduction

Mobile phone usage is becoming increasingly popular worldwide and consequently our exposure to the radiofrequency-electromagnetic radiation (RF-EMR) emitted by these devices is now unprecedented (1–3). Currently, the biological effects of this non-ionizing radiation remain the subject of active debate (4–7) and besides increasing reports of biological effects no robust clinical impacts have been established (8). Adding to the debate is the lack of a characterized mechanism by which RF-EMR could affect biological systems. There is broadly, two opposing bodies of evidence, some that support the potential of RF-EMR to elicit a suite of detrimental effects in a variety of cell and tissues types including altered brain nerve branching (9), oxidative stress (10–14), genotoxic assault highlighted by micronuclei formation (15, 16), and DNA fragmentation (13, 17, 18), while some find no elevation of oxidative stress (19–21) or DNA damage (22–24). The absence of a widely accepted mechanism of action that may account for the effects observed, complicates our ability to understand how RF-EMR interacts with specific cell types and to resolve the disparity in the literature.

Interest in the vulnerability of the male reproductive system to RF-EMR exposure has fueled an increasing number of recent studies. While such studies have yet to reach a firm consensus, they have revealed that sperm motility (18, 25, 26) and vitality (1, 13, 27) represent two key functional parameters that exhibit susceptibility to RF-EMR and can be significantly impaired following certain exposure regimes (28). Similarly, RF-EMR is capable of eliciting elevated reactive oxygen species (ROS) generation (13, 29–31), and also significant DNA fragmentation in spermatozoa (17, 18, 26, 32). In work conducted by our group (13), it was established that RF-EMR is capable of inducing oxidative stress in purified human spermatozoa. Hallmarks of this process included elevated generation of mitochondrial ROS that, in turn, resulted in impaired sperm motility and vitality, culminating in DNA fragmentation and oxidative DNA base adduct formation. Such results from cell culture studies may be viewed as highlighting the clinical importance of this area, given that the RF-EMR intensity eliciting these responses (1–2.8 W/kg) falls comfortably within the non-damaging exposure levels currently prescribed for this form of radiation (4 W/kg). Nevertheless, it is important to note that recent studies have revealed a level of variability in the responses documented following RF-EMR exposure. This variability may arise by virtue of the diverse exposure conditions employed in individual studies involving differing microwave intensity (SAR) and frequency (MHz/GHz), as well as variable exposure time (28), mode (continuous/intermittent) and method (waveguide/mobile phone device) of exposure (1). Notwithstanding these variations, a consistent theme may be emerging from studies reporting biological effects.

Oxidative stress is a major cause of defective sperm function, contributing to male infertility and DNA damage in the male germ line (33–36). Such a state of oxidative stress arises in spermatozoa predominantly as a result of increased ROS production from the mitochondria. The deleterious effects of excess ROS extend to the peroxidation of membrane lipids, generating cytotoxic aldehydes such as 4-hydroxynonenal (4HNE) (37, 38) and lead to the oxidation of DNA, generating adducts such as 8-hydroxy-2′-deoxyguanosine (8-OHdG) (39).

While many studies are now focusing on the biological effects of RF-EMR on reproductive systems, only four of these (17, 31, 40, 41) have investigated germ cell specific stages. In the current study, we have sought to extend the findings of our previous research (13) by focusing on whether key stages of germ cell development differ in their overall susceptibility to RF-EMR, seeking to uncover mechanism(s) that could account for any variability in response between the different cell types. Male germ cells present a key developmental model to utilize for studying the effects of RF-EMR during spermatogenesis. For this purpose, we employed cultured immortalized mouse germ cell lines (GC1, spermatogonial; GC2, spermatocyte) and cauda epididymal spermatozoa to determine the impact of RF-EMR exposure on immature germ cells and their mature counterparts. Using a similar experimental design to that reported in our previous study (13), these cells were exposed to RF-EMR in a waveguide for up to 6 h while being maintained at 23°C to mitigate any bulk thermal effects of this treatment. Following exposure, cells were assessed using a suite of functional assays to probe the potential impact of RF-EMR on oxidative stress in male germ cells, with a focus on the mitochondria as a potential source of RF-EMR-induced ROS generation.

## Materials and methods

### Chemical reagents

The chemicals and reagents used in this study were purchased from Sigma Aldrich (Sigma Chemical Co., St. Louis, MO, USA) unless stated otherwise, and were of research grade. The fluorescent probes were purchased from Invitrogen (Carlsbad, CA, USA) unless otherwise stated. Mouse germ cell lines were purchased from the American Tissue Culture Collection (ATCC; Rockville, MD, USA). These cell lines included type B spermatogonia-like GC1 (ATCC CRL-2053) and primary spermatocyte-like GC2 (ATCC CRL-2196) strains. Human embryonic kidney (HEK) 293 (ATCC CRL-1573), McCoy mouse fibroblast (ATCC CRL-1696) and COV434 human granulosa (Sigma Aldrich) cell lines were also used for comparison.

### Cell culture

All cell lines were grown at 37°C in 5% CO_2_, 95% air in Dulbecco's Modified Eagle's Medium (DMEM; ThermoFisher Scientific, Taren Point, NSW, Australia) supplemented with 100 mg/ml sodium pyruvate, 4.5 g/l glucose, 0.5 mM L-glutamine, 100 U/ml penicillin-streptomycin and 10% fetal bovine serum (ThermoFisher Scientific). For each experiment, cells were seeded to glass coverslips overnight at a concentration of ~1 × 10^5^ cells in 1 ml media in Greiner CELLSTAR multiwell culture plates (Sigma Aldrich). The passage number used was on average between 5 and 20, but this was maintained less than passage 25, where we observed no changes to proliferation or confluency. Cells were then subjected to EMR exposure for 0–6 h in DMEM media as described below.

### Primary cell and spermatozoa isolation

Primary spermatogonial germ cells were isolated from neonatal Swiss mice, as previously described (42). Testes were dissected, followed by removal of the tunica albuginea, washing in DMEM at 600 × g and 4°C for 5 min. Seminiferous tubules were then digested in 0.5 mg/ml collagenase for 15 min, resuspended in 0.5% v/v trypsin-EDTA and rotated for 15 min at 21°C. This sample was subsequently resuspended in DMEM and strained through a 70 μm filter to remove cell aggregates. The resulting suspension was layered above a continuous 2–4% BSA/DMEM gradient and allowed to sediment under gravity for 3 h to enrich for spermatogonia. The bottom 30 ml layer of this gradient was discarded and the second 40 ml layer, containing an enriched population of spermatogonia, was collected.

Studies undertaken with mouse spermatozoa followed experimental protocols approved by the University of Newcastle Animal Care and Ethics Committee (Ethics Number 2014-423). To isolate spermatozoa, epididymides were dissected from adult Swiss mice (>8 weeks) killed via CO_2_ asphyxiation. Mature spermatozoa were collected from the cauda epididymis by retrograde perfusion via the vas deferens (32, 43). The spermatozoa of three mice were utilized as independent replicates for each assay. These cells were resuspended at a concentration of 1 × 10^6^/ml in 1 ml of modified Biggers, Whitten, and Whittingham medium [BWW; (44)] in 35 mm petri dishes and, due to their short lifespan, were exposed to RF-EMR for up to a maximum of 4 h. Objective sperm motility was assessed by computer assisted sperm analysis (CASA; IVOS, Hamilton Thorne, Danvers, MA, USA). For this purpose, a minimum of 100 spermatozoa in five fields were assessed using 2X-CEL slides (Hamilton Thorne) suspended on a pre-warmed stage (37°C) (43). The following settings were utilized: negative phase-contrast optics, 60 frames/s recording rate, minimum cell size of 9 pixels, minimum contrast of 80, low size gate of 0.3, high size gate of 1.95, low intensity gate of 0.5, high intensity gate of 1.3, non-motile head size of 45 pixels, non-motile head intensity of 75, progressive average path velocity (VAP) threshold of 10 μm/s, slow (static) cells VAP threshold of 5 μm/s, slow (static) cells straight-line velocity (VSL) threshold of 0 μm/s, and threshold straightness (STR) of 75%. Cells exhibiting a VAP of >10 μm/s and a STR >0 were considered progressive. Cells with a VAP greater than that of the mean VAP of progressive cells were considered rapid. Sperm vitality was assessed via the eosin exclusion method (45).

### EMR waveguide exposure system

Cells were exposed to EMR in a waveguide apparatus emitting radiofrequency, continuous waves produced by a SMC100A signal generator (Rohde and Schwarz, Macquarie Park, NSW, Australia). The signal intensity was adjusted to appropriate levels with a signal amplifier as used by De Iuliis et al. (13) and output was split through a network antenna to direct the RF-EMR to the aluminum exposure cage, and with minimal wave discharge to a spectrum analyzer to assess the radiation levels (Advantest, Tokyo, Japan). RF-EMR reflection within the cage was minimized by addition of carbon-impregnated foam (RFI Industries, Bayswater, VIC, Australia) around the exposure setting. As a safety precaution, microwave generation was only initiated when the lid was securely placed on the exposure chamber. Furthermore, external safety testing was conducted to ensure no leakage of microwave radiation. Microwaves were generated at a frequency of 1.8 GHz and intensity of 0.15 or 1.5 W/kg specific absorption rate (SAR) as previously calculated by De Iuliis et al. (13). For this study the calibration procedure utilized a fiber optic thermometer with an “STF” probe (Luxtron, LumaSense Technologies) to measure temperature rises during irradiation. In addition, the uniformity of the radiation across the petri dish was checked using the computer code CST Microwave Studio (www.cst.com), to simulate the irradiation and append SAR calculations. For exposure, germ cells seeded to coverslips or spermatozoa were situated in a small petri dish inside the apparatus. Untreated controls were placed outside of the Faraday cage of the unit and were maintained under identical environmental conditions, in the dark at 23°C. The temperature of these media was measured over the course of the experiments, with no significant fluctuations observed in both exposed and control samples, with a stable reading of 23°C (±0.2°C; Supplementary Figure 1D) observed.

### Alkaline comet assay

The Comet assay was performed as detailed by Katen et al. (46, 47). Germ cells and spermatozoa were pelleted and stored at −80°C before being resuspended in phosphate buffered saline (PBS) at a concentration of 4 × 10^4^ cells/μl. A 10 μl sample of this cell suspension was mixed with 70 μl agarose (Trevigen, Gaithersburg, MA, USA) and allowed to set on Dakin G376 slides pre-coated with 1% low melting point agarose (ProSciTech, Kirwan, QLD, Australia) sealed with a coverslip overnight at 4°C. After removing the coverslip, slides were treated with lysis solution 1 [pH 7.5; 0.8 M Tris–HCl, 0.8 M dithiothreitol [DTT], 1% SDS; (48)] and sealed with a coverslip for 30 min, followed by lysis solution 2 (pH 7.5, 0.4 M Tris–HCl, 50 mM EDTA, 2 M NaCl, 0.4 M DTT) under the same conditions. Again, coverslips were removed and slides were washed in tris-boric acid-EDTA (TBE) solution (0.445 M Tris–HCl, 0.445 M boric acid, 10 mM EDTA) for 10 min. In preparation for electrophoresis, slides were treated with alkaline solution (pH 11.5; 0.03 M NaOH, 1 M NaCl) for 15 min at 4°C, followed by electrophoresis in alkaline buffer (pH 12; 0.03 M NaOH) for 4 min at 1 V/cm. To neutralize the assay, slides were washed in neutralization solution (pH 7.5; 0.4 M Tris–HCl) for 5 min. SYBR green nucleic acid stain (ThermoFisher Scientific, Taren Point, NSW, Australia) (diluted to 1 × from a 10,000 × stock solution in 10 mM Tris/PBS) was applied to the slides immediately before viewing on the microscope, and a coverslip was added. Slides were imaged with a Zeiss Axioplan 2 fluorescence microscope (Carl Zeiss MicroImaging Inc., Kirchdorf, Germany), and the integrity of the cells was analyzed using Comet Assay IV software (Perceptive Instruments, Suffolk, UK). A minimum of 30 cells were analyzed for GC1/GC2 cells per replicate, with 3 replicates completed. Twenty to fifty cells were analyzed for sperm samples per replicate, with 3 replicates completed.

### Oxidative DNA damage assay (8-OHDG)

In order to determine the level of 8-OHdG DNA base adduction following RF-EMR exposure, DNA was extracted from GC1, GC2, and sperm cells by the phenol/chloroform method. A sample of 5 × 10^6^ cells were suspended in 1 ml STE buffer (500 mM NaCl, 100 mM Tris-HCl [pH 8], 10 mM EDTA) and were supplemented with 50 μl 20% SDS, 10 μl 2-mercaptoethanol and 100 μl 20 mg/mL proteinase K (Roche, Castle Hill, NSW, Australia). After overnight incubation at 55°C, an equal volume of phenol:chloroform:isoamylalcohol (25:24:1) was added to each tube and vortexed for 30 s. Each tube was then centrifuged at 14,000 × g for 15 min. The top layer of each sample was collected and transferred to a new 1.5 ml Eppendorf tube. Sodium acetate (3 M) was then added at a volume of 1/9 in addition to two volumes of ice-cold 100% ethanol. The tubes were mixed by inversion and placed at −20°C overnight. Following this, the DNA was pelleted by centrifugation at for 15 min at room temperature. Next, the supernatant was decanted and the pellet was washed with 100 μl 70% ethanol to remove salts. Finally, the DNA was pelleted by centrifugation at 14,000 × g for 15 min, air dried and resuspended in water. DNA concentration was revealed via spectrophotometry at 260 nm and quantification of 8-OHdG formation was then performed with an 8-OHdG ELISA kit (Abcam, Cambridge, UK). The ELISA plate was developed in the dark on an orbital shaker for 60 min before being read on a Fluostar Optima plate reader (BMG Labtech, Mornington, Victoria, Australia) at a wavelength of 405–10 nm.

### Sperm chromatin dispersion (Halo) assay

The halo assay is a qualitative method to assess DNA integrity of spermatozoa, whereby cells treated with DTT and SDS will have their DNA splay out, if intact. The DNA is then stained with 4′,6-diamidino-2-phenylindole (DAPI) to visualize a halo-like pattern (49). Cells snap frozen and stored at −80°C were mixed with 1% low melting agarose at 37°C to achieve a concentration of 0.7% agarose. A 70 μl aliquot of this solution was then transferred to a Superfrost microscope slide precoated with 0.65% agarose, sealed with a coverslip and placed at 4°C for 5 min to solidify. Following this, the coverslip was gently removed and the slides were treated with 0.08 N HCl for 7 min in the dark. The slides were then treated with halo solution 1 (pH 7.5; 0.4 M tris, 1% SDS, 50 mM EDTA, 0.8 M DTT) for 10 min, followed by halo solution 2 (pH 7.5; 0.4 M tris, 1% SDS, 2 M NaCl) for 5 min at room temperature to lyse the cells, relax and neutralize the DNA. Next, the slides were treated with tris-boric acid-EDTA buffer (pH 7.5; 0.1 M tris, 0.09 M boric acid, 0.002 M EDTA) for 2 min, followed by washes in increasing concentrations of 70, 90, and 100% ethanol for 2 min each, to dehydrate the slides. The slides were allowed to air dry before staining with DAPI for 10 min at room temperature. Finally, the slides were rinsed in PBS and mounted for viewing with a Zeiss Axioplan 2 fluorescence microscope (Carl Zeiss). A minimum of 100 cells were assessed for the number of intact halos.

### Assessment of germ cell mitochondria following RF-EMR exposure

Coverslips containing seeded, exposed cell lines were incubated (15 min at 37°C) in the dark in 50 μl droplets of DMEM containing 1 μM MitoSOX Red (MSR) to detect mitochondrial superoxide generation and 5 nM SYTOX green stain for assessment of cell vitality. Following incubation, coverslips were washed in DMEM and mounted in 5 μl DMEM on microscope slides. A minimum of 100 cells were then assessed using a Zeiss Axioplan 2 fluorescence microscope. In order to discriminate the potential mitochondrial origin of ROS under RF-EME exposure, we utilized a previously published strategy, which selectively impedes electron flow at discrete stages of the electron transport chain (ETC) (50). For experiments in which mitochondrial electron transfer was inhibited in this fashion, seeded coverslips were treated with a final concentration of 10 μM of either antimycin A (complex III inhibitor) or rotenone (complex I inhibitor) for 0–6 h at 21°C and again assayed with the MSR probe, as detailed above. Finally, for experiments involving succinate as the electron source, germ cells were seeded overnight in DMEM described above, and refreshed with DMEM, or DMEM devoid of glucose containing 5 mM succinate, for the duration of the experiment (4 h). The purpose of using a glucose free medium is to force electrons into the ETC via Complex II with the use of succinate as an electron donor.

### Determination of sperm oxidative stress following RF-EMR exposure

Spermatozoa were used for determination of mitochondrial ROS generation (MSR), mitochondrial membrane potential (MMP), lipid peroxidation (BODIPY C11), and protein tyrosine phosphorylation level (α-PT66) using a FACS-Canto flow cytometer (BD Biosciences, San Jose, CA, USA) equipped with a 488 nm argon laser and 633 nm helium-neon laser. Gating was used to prevent incorporation of non-sperm cells and the evaluations were based on at least 5,000 gated cells. Regarding the MSR and JC1 assays, sperm cells were centrifuged at 450 × g for 5 min and resuspended in a final concentration of 500 nM MSR or JC1 coupled with a 5 nM SYTOX green vitality stain. Incubation of this probe was for 15 min at 37°C in the dark, followed by resuspension in BWW media in flow cytometry tubes for analysis on the flow cytometer. The BOPIDY probe was preloaded at 5 μM for 1 h at 37°C. Cells were then washed and treated, with arachidonic acid (50 μM) employed as a positive control. Analysis of these data was undertaken using CellQuest software (BD Biosciences, San Jose, CA, USA).

Chemiluminescence was used to investigate hydrogen peroxide generation in treated populations of germ cells and spermatozoa as previously described (51). Briefly, 2 × 10^6^ cells were resuspended in BWW supplemented with 4 μl of 250 μM luminol and 8 μl of 2 mg/ml horseradish peroxidase (HRP, type VI from horseradish) in a total of 400 μl BWW. These samples were assessed for chemiluminescence in Rohren tubes (Sarstedt, Numbrecht, Germany) for 2 h at 37°C in a Berthold 9505C luminometer (Berthold, Wilbad, Germany). Control Version 1.04B was used for the system software.

### Statistical analysis

JMP version 11 (SAS Institute Inc., Cary, NC) was used to analyze the data in each experiment, which were performed with at least 3 independent replicates. EMR treatment was analyzed using a one-way ANOVA at each time point, paired with Tukey's honest significant difference test (significance *p* < 0.05). Error bars are presented as standard error values around the mean.

## Results

### Mouse male germ cells are vulnerable to RF-EMR

**Figure 1:**
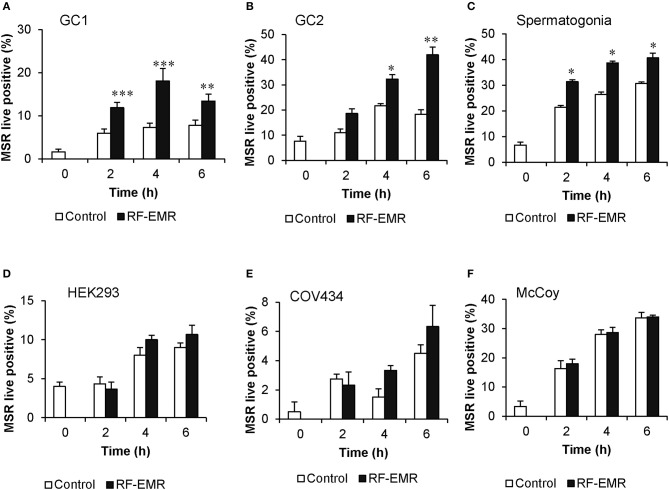
RF-EMR exposure (1.8 GHz, 0.15 W/kg) induces mitochondrial superoxide generation in male germ cells. **(A)** Spermatogonia-like (GC1) cell line, **(B)** spermatocyte-like (GC2) cell line, and **(C)** spermatogonia isolated from neonatal mice were seeded to glass coverslips overnight and exposed to RF-EMR (1.8 GHz, 0.15 W/kg) for periods of up to 6 h. Somatic cell lines comprising **(D)** human embryonic kidney cells (HEK293), **(E)** granulosa cells (COV434) and **(F)** mouse fibroblasts (McCoy), were treated under an identical exposure regime (1.8 GHz, 0.15 W/kg) as negative controls. At regular intervals during exposure, a portion of the cells were assessed for mitochondrial ROS production using the MitoSOX red (MSR) probe. This analysis was restricted to the live cell population as determined by co-labeling with SYTOX green vitality stain. These analyses were performed on at least three biological replicates and data are presented as mean ± SEM. ***p < 0.001, **p < 0.01, *p < 0.05 compared to unexposed controls.

Cell lines representing both spermatogonial (GC1) and spermatocyte (GC2) phases of development exposed to RF-EMR at a dose of 0.15 W/kg exhibited significant increases in the formation of mitochondrial ROS following 2 h (*p* < 0.001) and 4 h (*p* < 0.05) of exposure, respectively (Figures 1A,B). This phenomenon persisted up to the 6 h time point for both cell types (*p* < 0.01). Furthermore, as shown in Figure 1C, this result was recapitulated in populations of primary spermatogonial cells isolated from neonatal mice. Here, we again observed significantly elevated mitochondrial ROS generation, after 2, 4, and 6 h of exposure (*p* < 0.05) compared to unexposed control populations. In these primary cultures, we again observed no effect of RF-EMR exposure on vitality. While we documented a modest decrease in vitality after 6 h from the initial assessment 93% ± 0.7, RF-EMR exposure did not significantly decrease this measure (88% ± 1.1) with respect to the untreated control spermatogonia (83% ± 2.9) within this period. An identical RF-EMR treatment regime failed to elicit any overt changes in mitochondrial ROS generation (MitoSOX labeling) of the three somatic cell lines examined (Figures 1D–F; HEK293, COV434, and McCoy, respectively) beyond that of the untreated control samples. In both GC1 and GC2 cell lines, ROS generation was not notably increased following exposure with an elevated dose of 1.5 W/kg EMR (Supplementary Figures 1A,B) compared to the dose of 0.15 W/kg. Importantly, the effects of exposure were generated independent of any significant reduction in cell viability, in all cell types and treatment regimes employed in this study (Supplementary Figure 2).

### The origin of EMR mediated ROS generation in male germ cells

**Figure 2:**
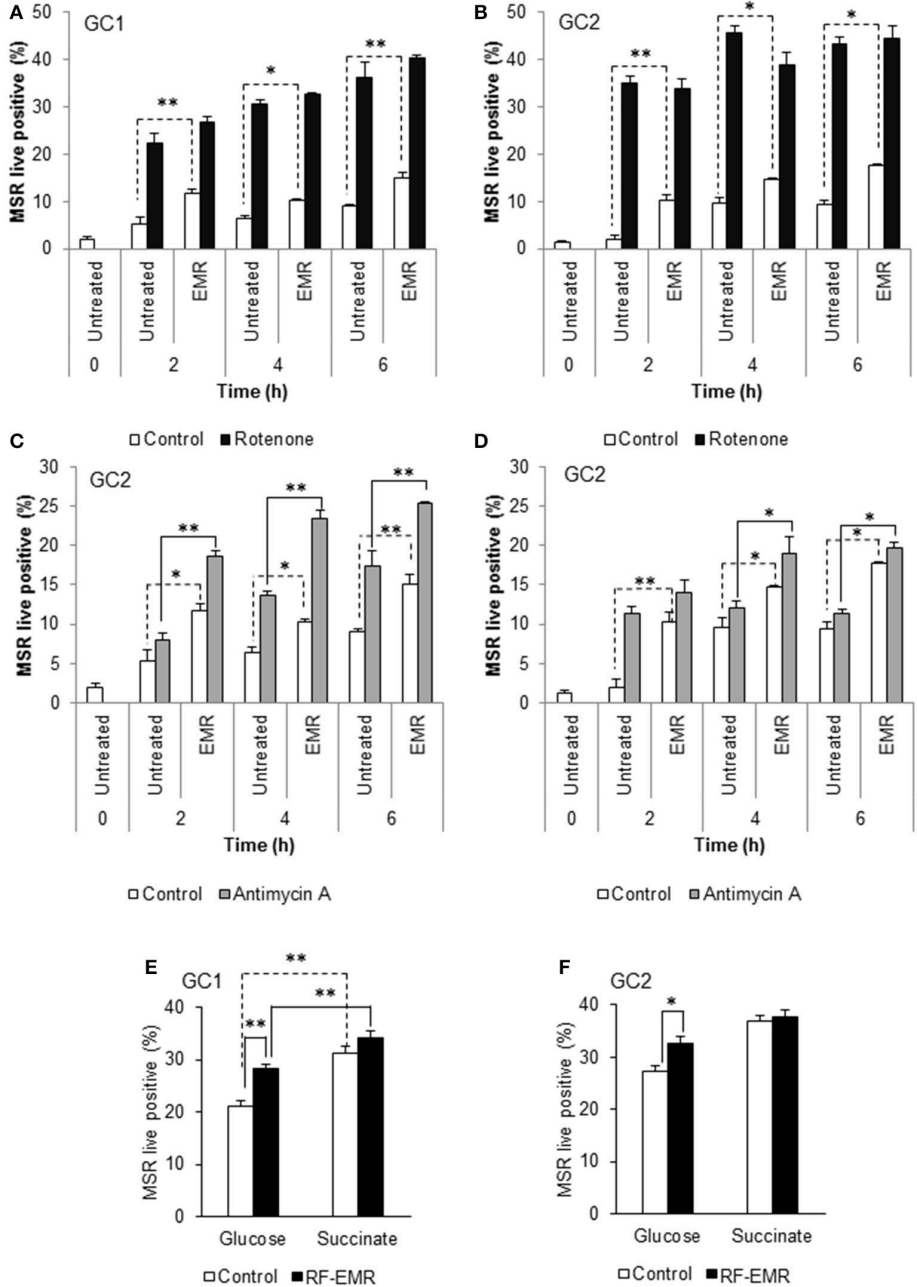
Inhibition of mitochondrial respiration in the presence of RF-EMR is associated with increased ROS production. **(A)** Spermatogonia-like (GC1) and **(B)** spermatocyte-like (GC2) cell lines were seeded to glass coverslips overnight and treated with mitochondrial electron transport chain inhibitor rotenone, in the presence or absence of RF-EMR exposure (1.8 GHz, 0.15 W/kg), for periods of up to 6 h. Alongside these experiments, another electron transport inhibitor, antimycin A, was also utilized for GC1 **(C)** and GC2 cells **(D)**. Mitochondrial ROS production was assessed using the MSR probe. This analysis was again restricted to the live population, determined by co-labeling with SYTOX green vitality stain. Glucose and succinate substrates were utilized for comparison of mitochondrial ROS generation in GC1 **(E)** and GC2 **(F)** cells in the presence of RF-EMR. Cells were seeded to coverslips overnight in DMEM media as detailed above, and refreshed with this DMEM media or 5 mM succinate media DMEM (devoid of glucose) for the course of the experiment. These analyses were performed on at least three biological replicates and data are presented as mean ± SEM. **p < 0.01, *p < 0.05 compared to unexposed controls.

Following the demonstration that GC1 cells responded to EMR exposure in a similar manner to primary spermatocytes, we focused our characterization of downstream effects of EMR on the GC1 and GC2 cell lines. Given that the mitochondria account for a majority of ROS production within the mature spermatozoon (34, 38) and the observed increase in mitochondrial ROS generation following germ cell exposure to RF-EMR (Figures 1A,B), we next investigated the effects of treating GC1/GC2 germ cell lines with a combination of RF-EMR and inhibitors that selectively target either Complex I or III of the ETC (Figure 2). This study demonstrated that inhibition of Complex I with rotenone had a marked impact on both CG1 and CG2 cell types, dramatically increasing ROS production via mechanisms that were independent of RF-EMR exposure (Figures 2A,B). In contrast, while inhibition of Complex III with antimycin A predictably induced a significant increase in mitochondrial ROS generation in the GC1 cell line alone (Figure 2C; *p* < 0.01), under the exposure regime this inhibitor did significantly potentiate the impact of RF-EMR exposure in both cell lines (Figures 2C,D; *p* < 0.01). Accordingly, after 2 h antimycin A treated GC1 cells were characterized by ROS levels that were significantly elevated above that of non-exposed cells (Figure 2C; *p* < 0.01). A similar, although delayed, response was also recorded in GC2 cells, with significance differences in mitochondrial ROS generation only being achieved after a period of 4 h (Figure 2D; *p* < 0.05). To aid in pinpointing the components of the mitochondrial ETC vulnerable to RF-EMR, succinate was employed as a metabolic substrate; driving electrons to enter this pathway via Complex II. In GC1 cells (Figure 2E) the introduction of this metabolic substrate elevated basal ROS levels substantially in both control (*p* < 0.01) and RF-EMR (*p* < 0.01) treated cells. However, RF-EMR exposure did not induce a significant increase in mitochondrial ROS generation when utilizing succinate as substrate as mirrored in GC2 cells (Figure 2F). This was not the case when both GC1 (Figure 2E) and GC2 (Figure 2F) cells were sustained with glucose, with RF-EMR exposure elevating mitochondrial ROS significantly (*p* < 0.01 and *p* < 0.05 respectively).

**Figure 3:**
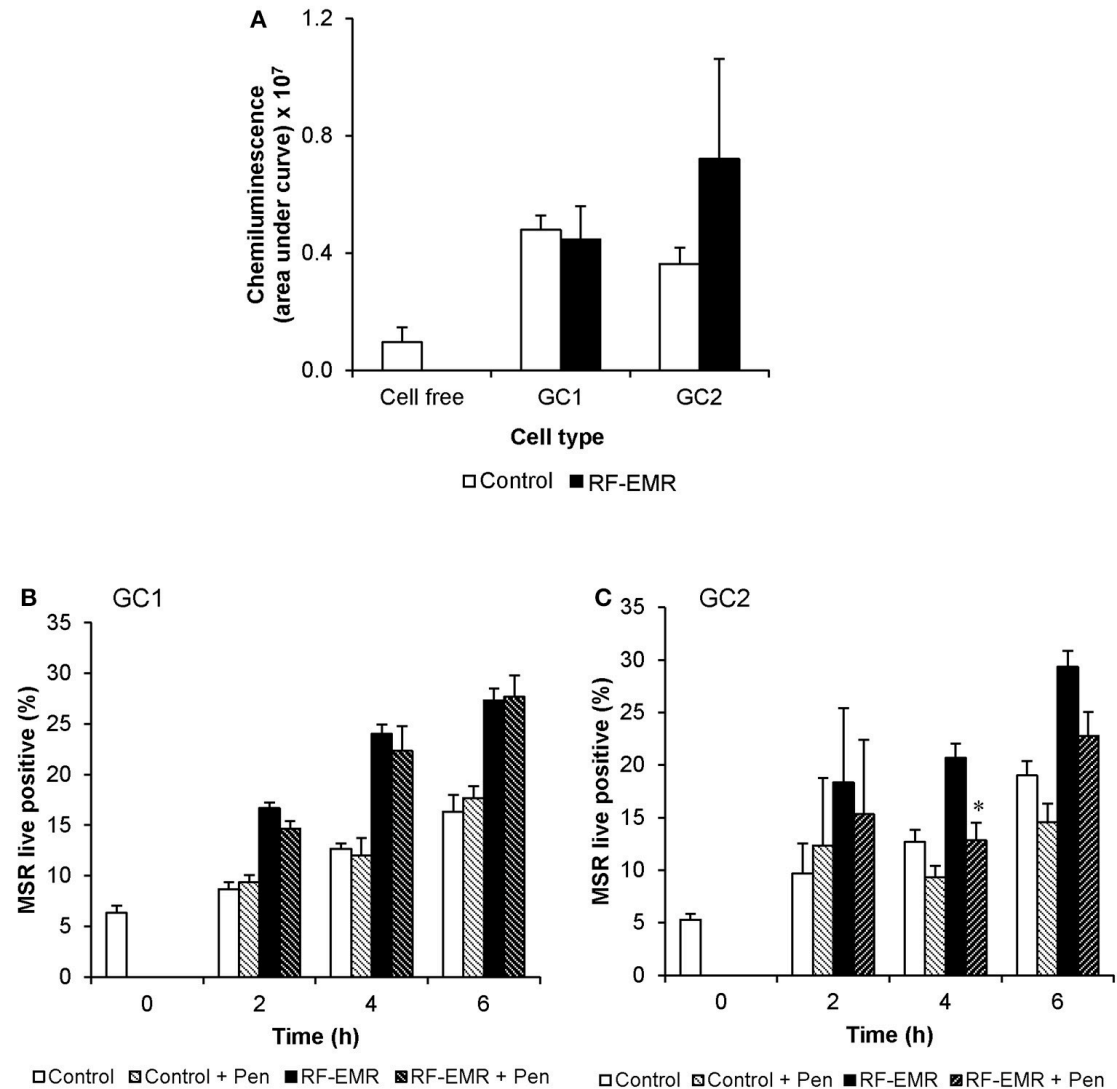
The effects of RF-EMR on production of reactive oxygen species and lipid peroxidation in male germ cells. GC1 and GC2 cells were seeded to coverslips overnight then exposed to RF-EMR of an intensity of 0.15 W/kg and frequency of 1.8 GHz. **(A)** Luminol-peroxidase chemiluminescence assessment of ROS production was conducted on populations of GC1 and GC2 cells following 6 h of exposure. The ability of penicillamine (100 μM), a potent aldehyde scavenger, to prevent RF-EMR induced ROS production was also assessed over the course of exposure in both **(B)** GC1 and **(C)** GC2 cells. These analyses were performed on at least three biological replicates and data are presented as mean ± SEM. *p < 0.05 compared to RF-EMR treatment.

In order to determine if the increase in mitochondrial ROS in the irradiated germ cells could advance cellular oxidative stress as shown in previous studies (1, 13, 52), we next investigated markers of cellular ROS production and lipid peroxidation following application of RF-EMR. Examination of extracellular ROS production using a luminol-peroxidase chemiluminescence assay revealed no significant increases in the release of ROS from these cells following RF-EMR exposure (0.15 W/kg) for any of the mouse germ cell types examined (Figure 3A). Penicillamine blocks damaging intracellular alkylation events that are mediated by lipid peroxidation by-products, inhibiting the propagation of oxidative stress therefore promoting an antioxidant effect (53). Incorporation of this potent nucleophile scavenger under our exposure conditions, provided no significant reduction in the elevated levels of ROS production observed in RF-EMR treated GC1 cells (Figure 3B). While a similar response to RF-EMR was observed in GC2 cells (Figure 3C), penicillamine treatment did achieve a significant decrease in ROS originating from the mitochondria following 4 h of exposure (*p* < 0.05). However, this observation did not persist to the 6 h time point.

### RF-EMR does not induce significant DNA damage in male germ cell lines

**Figure 4:**
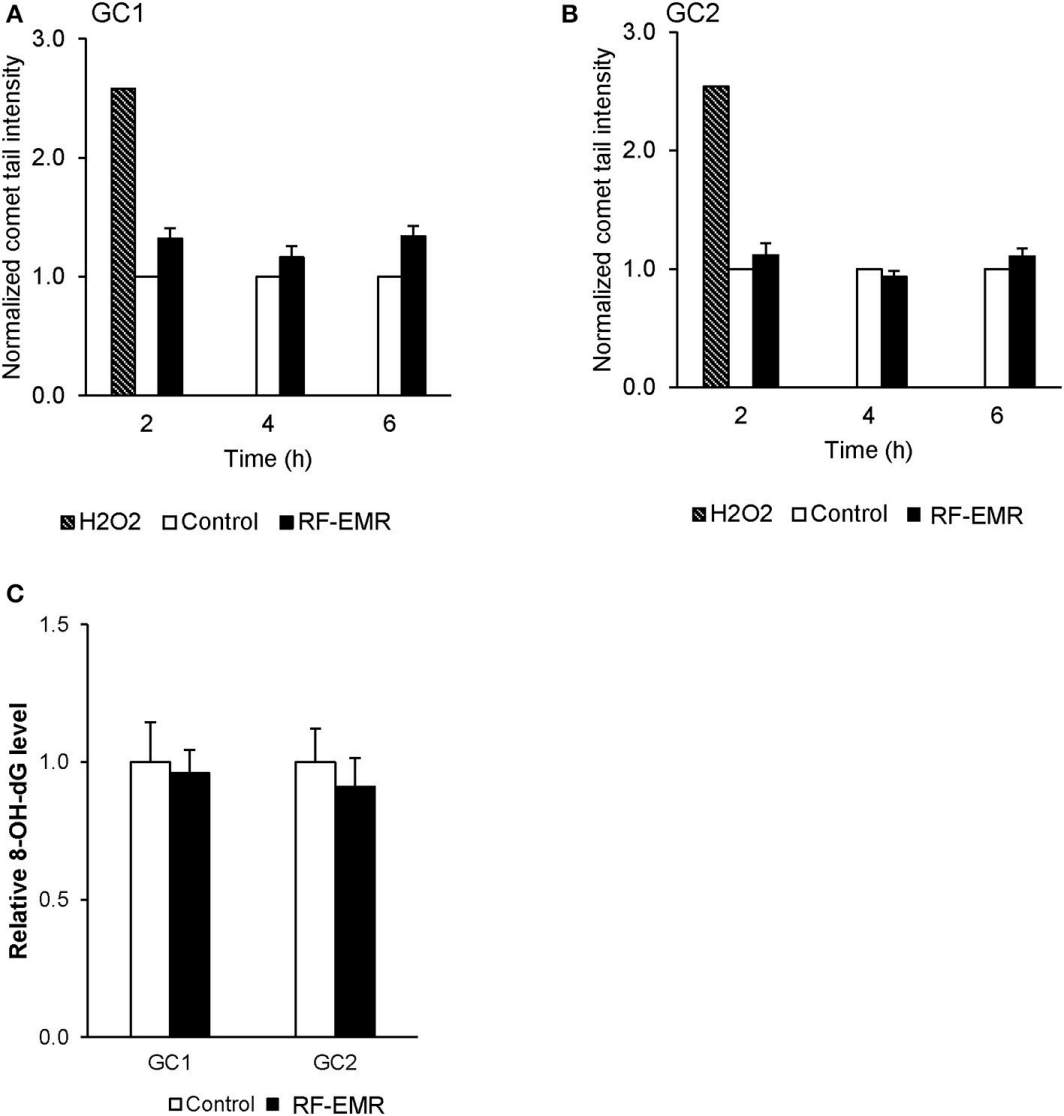
The effect of RF-EMR on DNA fragmentation and DNA oxidation within mouse germ cells. GC1 and GC2 cell lines seeded to glass coverslips overnight were exposed to RF-EMR (1.8 GHz, 0.15 W/kg) to determine its ability to impair DNA integrity. These cells were subsequently assessed for DNA fragmentation using an alkaline comet assay; **(A)** GC1, **(B)** GC2. From cells exposed for 6 h, DNA was extracted via the use of phenol-chloroform methodology in order to assess oxidative DNA damage in the form of 8-hydroxy, 2-deoxyguanosine (8-OHdG) base adducts, as evaluated by an 8-OHdG ELISA **(C)**. These analyses were performed on at least three biological replicates.

To confirm the potential of RF-EMR to induce genotoxic effects in male germ cells [as documented in previous studies (17, 41)] we next investigated the incidence of DNA strand breakage, utilizing the alkaline comet assay (Supplementary Figure 1E). Here, it was revealed that RF-EMR did not induce significant DNA fragmentation in GC1 cells (*p* = 0.07) following 6 h exposure (Figure 4A), with even less evidence for damage evident in GC2 cells, over all time points examined (Figure 4B). Furthermore, to confirm the lack of DNA damage observed we investigated the presence of the oxidative DNA base adduct, 8-OHdG, in GC1 and GC2 cells (Figure 4C). In keeping with the lack of DNA damage and the inability of RF-EMR to induce lipid peroxidation (Figure 4), this exposure induced no significant increases in the generation of 8-OHdG in either germ cell line population (*p* > 0.1).

### The effects of RF-EMR on mature mouse spermatozoa

**Figure 5:**
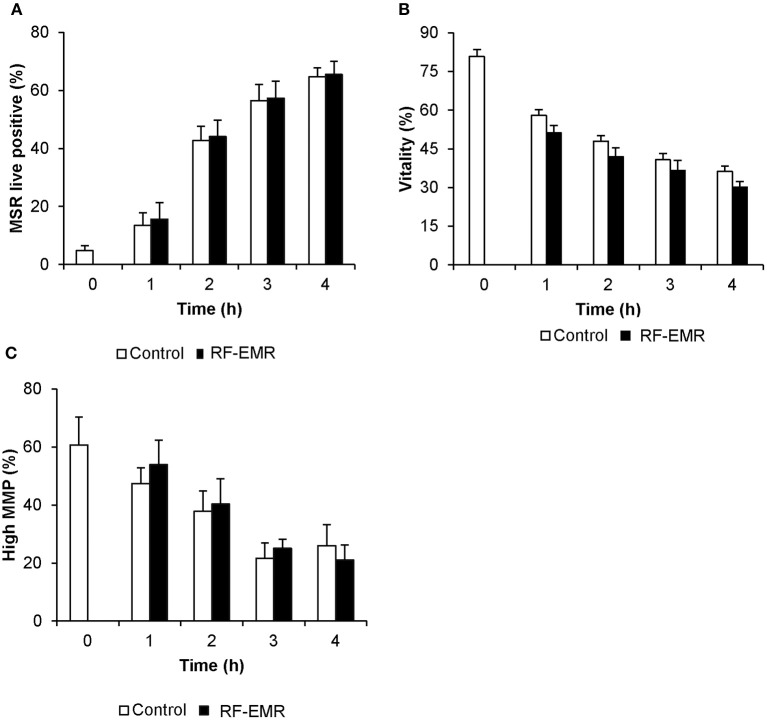
Susceptibility of mouse spermatozoa to RF-EMR (1.8 GHz, 0.15 W/kg). Mature mouse spermatozoa isolated from the cauda epididymis were exposed to RF-EMR of an intensity of 0.15 W/kg. At regular intervals during exposure, a portion of the live cell population was assessed for **(A)** mitochondrial ROS generation using the MSR probe via flow cytometry. These cells experienced a highly significant time dependent increase in ROS production (*p* < 0.001). **(B)** Similarly, total vitality was evaluated with an eosin stain. **(C)** Alternatively, perturbation of mitochondrial membrane potential (MMP) was determined through incubation with the JC1 probe. In this instance, the percentage of cells displaying green fluorescence indicative of high mitochondrial membrane potential was determined, again via flow cytometry. Both vitality and MMP measures experienced significant time-dependent decreases independent of RF-EMR exposure (*p* < 0.001). These analyses were performed on at least three biological replicates and data are presented as mean ± SEM.

In marked contrast to the response elicited by RF-EMR in germ cell lines and purified spermatogonia, mature populations of mouse spermatozoa sampled from the cauda epididymis proved refractory to this exposure. In this regard, we failed to detect any substantive increase in either mitochondrial ROS (Figure 5A) or cell death (Figure 5B) following exposure of spermatozoa to a dose of 0.15 W/kg RF-EMR. Rather, these terminally differentiated cells exhibited a spontaneous highly significant (*p* < 0.001) and time-dependent increase in ROS generation in association with sperm capacitation that was not influenced by RF-EMR exposure (Figure 5A). These changes were accompanied by significant, time-dependent reductions in sperm vitality (Figure 5B; *p* < 0.001) and MMP (Figure 5C; p < 0.001) that were again uninfluenced by exposure to RF-EMR. Increasing the intensity of this radiation to 1.5 W/kg (Supplementary Figure 1C) resulted in a modest reduction in ROS generation after 1 h (*p* < 0.05) of exposure. This trend of reduced ROS production was held over the ensuing 3 h incubation, however, did not maintain significance (*p* = 0.11).

**Figure 6:**
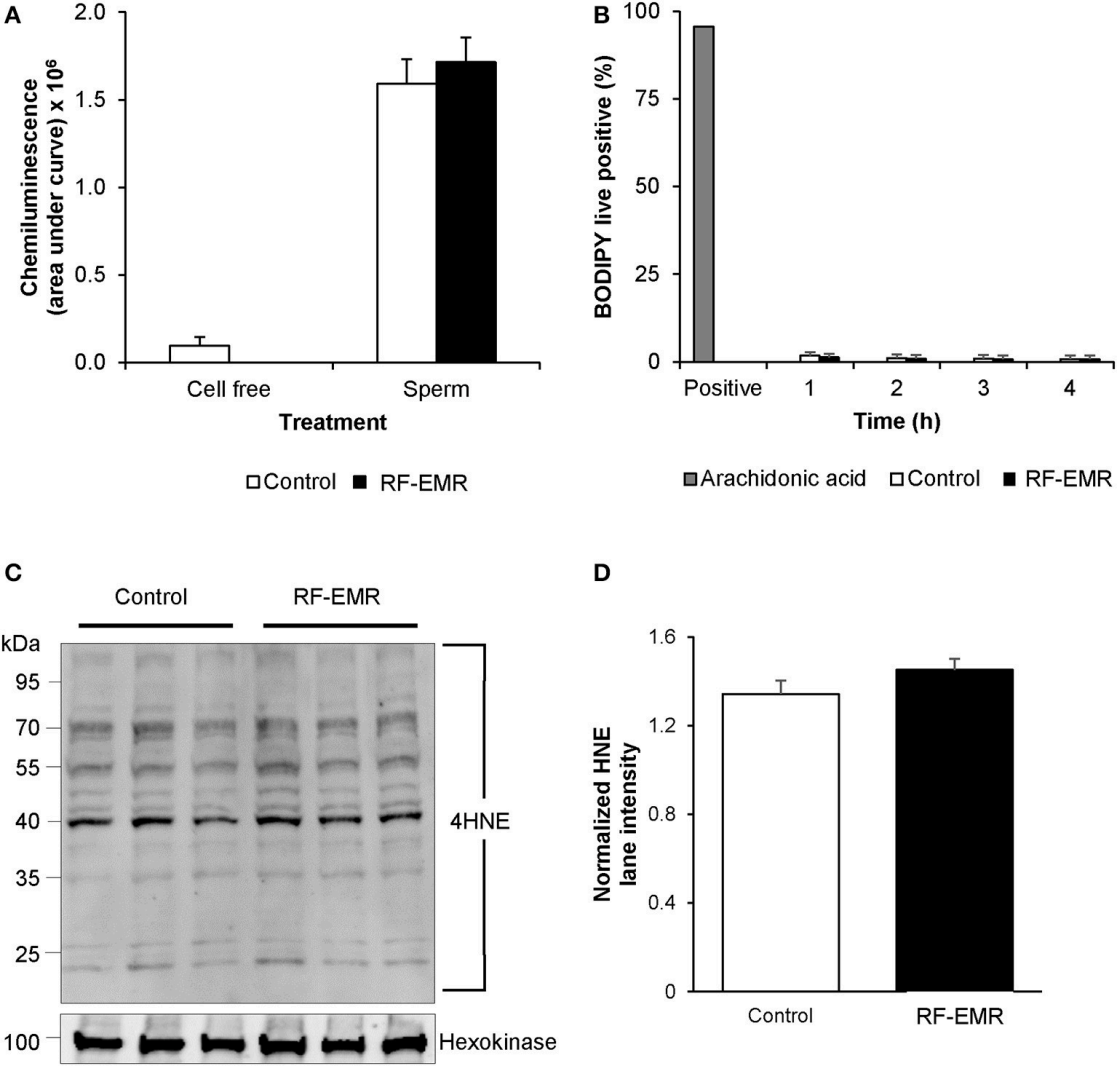
The effects of RF-EMR on production of reactive oxygen species and lipid peroxidation in mature spermatozoa. Spermatozoa were isolated from the cauda epididymis and exposed to RF-EMR of an intensity of 0.15 W/kg. **(A)** Luminol-peroxidase chemiluminescence was used to assess ROS production following 4 h of exposure. **(B)** The lipid peroxidation status of these spermatozoa was evaluated with the BODIPY C11 probe via flow cytometry. **(C)** The profile of 4HNE alkylated-proteins in RF-EMR exposed spermatozoa was assessed via immunoblotting, with hexokinase expression featuring as a loading control. Three replicates were performed for both control and RF-EMR treated sperm protein extracts. **(D)** The corresponding intensity of all 4HNE labeled protein bands extracted from untreated control, and RF-EMR exposed spermatozoa, was determined by densitometric analysis of pixel intensity. Densitometry was performed on the principal bands from 70 to 40 kDa, relative to the 100 kDa hexokinase band presented below the 4HNE immunoblot (full blot shown in Supplementary Figure 1F).

Again, utilizing chemiluminescence, global cellular ROS generation in mature mouse spermatozoa was unaffected following exposure to RF-EMR (Figure 6A). Accordingly, the levels of lipid peroxidation associated with spermatozoa, as assessed using the BODIPY C11 probe (and arachidonic acid positive control), were also not significantly elevated following RF-EMR exposure (Figure 6B). Furthermore, both the qualitative profile and relative levels of 4-hydroxynonenal-alkylated sperm proteins remained unchanged with exposure to RF-EMR (Figures 6C,D).

### RF-EMR induces DNA damage in mature mouse spermatozoa

**Figure 7:**
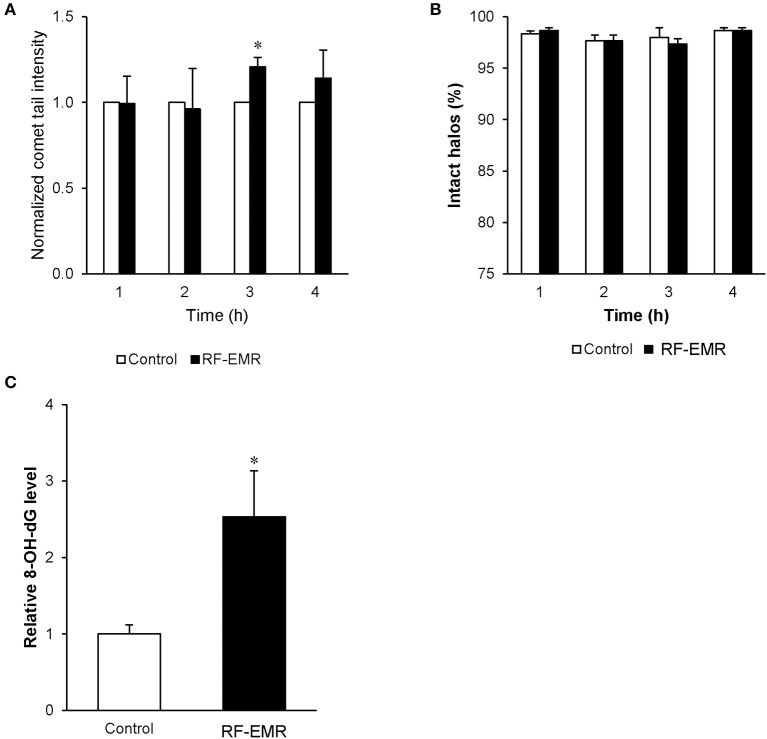
The effect of RF-EMR on DNA oxidation and fragmentation within mature sperm cells. Mature cauda mouse spermatozoa were exposed to RF-EMR (0.15 W/kg) to determine its ability to impair DNA integrity. These spermatozoa were subsequently assessed for DNA damage using the **(A)** alkaline comet assay **(B)** and halo assay. **(C)** From cells exposed for 4 h, DNA was extracted via the use of phenol-chloroform and analyzed for the presence of oxidative DNA damage utilizing an 8-OHdG ELISA. *p < 0.05 compared to unexposed controls.

In order to determine if spermatozoa were also sensitive to DNA damage following RF-EMR exposure we repeated our alkaline comet assay in these mature cells (Figure 7A). This assay demonstrated a significant 20% increase in DNA fragmentation in RF-EMR treated spermatozoa following 3 h of exposure (*p* < 0.05). Again, this trend of DNA damage was observed after 4 h of exposure but did not maintain significance. We next utilized a complementary DNA damage assay (halo; Figure 7B); however, no concomitant reduction in DNA integrity in response to RF-EMR exposure was observed with this assay. While little evidence of excess ROS production could be observed in real time in these mature gametes, hallmarks of oxidative stress states were observed with elevated expression of oxidative DNA lesions (8-OHdG) present within RF-EMR treated spermatozoa, following 4 h of radiation exposure (Figure 7C).

### Functional consequences of RF-EMR exposure in mouse spermatozoa

**Figure 8:**
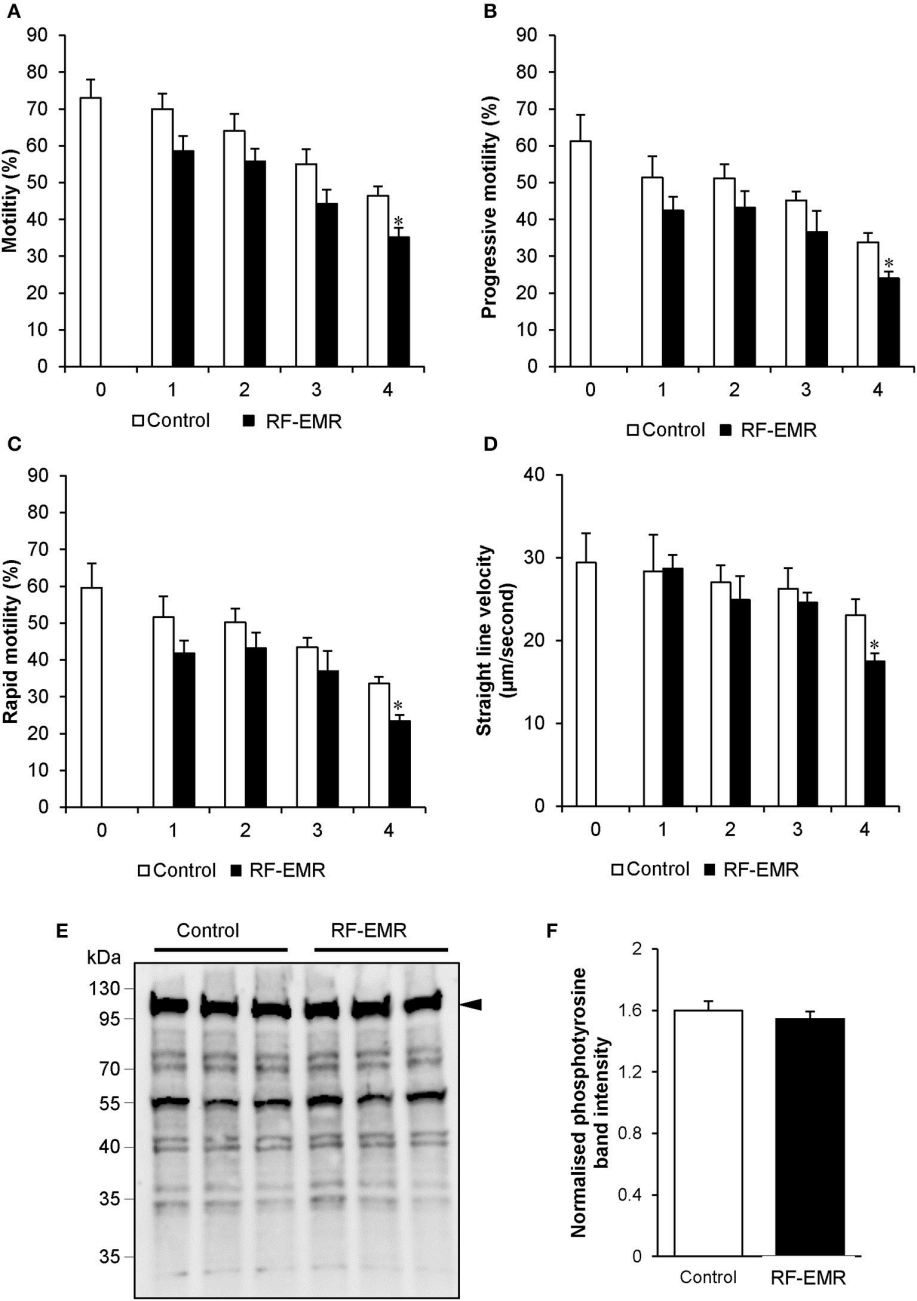
RF-EMR alters sperm motility but not tail tyrosine phosphorylation levels. Mature mouse spermatozoa isolated from the cauda epididymis were exposed to RF-EMR of an intensity of 0.15 W/kg for periods of up to 4 h. At regular intervals during exposure, computer assisted sperm analysis was performed for parameters of sperm motility. **(A)** Sperm total, **(B)** progressive, **(C)** rapid motility, and **(D)** straight-line velocity. **(E)** Spontaneous sperm tyrosine phosphorylation levels were assessed via immunoblotting. Three replicates were performed for both control and RF-EMR treated sperm protein extracts. The intensity of each lane was then quantified via pixel intensity **(F)**. The entire lane was quantified relative to hexokinase, as the loading control (arrow). These analyses were performed on at least three biological replicates. *p < 0.05 compared to unexposed controls.

Considering RF-EMR did not induce marked alterations to mitochondrial function in spermatozoa, but was capable of inducing DNA fragmentation and oxidative DNA damage in this cell type, we next explored its effect on basic sperm physiology. The modest but significant DNA fragmentation induced at 3 h of RF-EMR exposure (Figure 7A) was not accompanied by any observable impacts on sperm function, with spermatozoa retaining motility profiles that were indistinguishable from that of untreated control samples (Figures 8A–D). However, at 4 h of exposure significant impacts were observed. A significant decrease in total sperm motility (46 vs. 35%; *p* < 0.05), reductions in progressive motility (*p* < 0.05; Figure 8B), rapid motility (*p* < 0.05; Figure 8C), and the straight line velocity (*p* < 0.05; Figure 8D) of exposed spermatozoa, were all observed at the 4 h time point. These effects of RF-EMR exposure were not associated with any change in the capacitation status of the spermatozoa as reflected in their patterns protein phosphotyrosine expression, which remained uniformly high (Figure 8E).

## Discussion

In this study we have explored the effects of RF-EMR on both cultured male germ cell lines and spermatozoa isolated from the mouse. Our results align with previous studies in this field, which demonstrate the capacity of RF-EMR to induce DNA strand breakage, mitochondrial free radical generation and motility loss. A modest but clear increase in mitochondrial ROS production in our germ cell models points toward an oxidative stress pathological mechanism of RF-EMR. The response of cultured spermatozoa to RF-EMR was dissimilar, with no overt active ROS production observed when these cells were probed directly after exposure. Nevertheless, hallmarks of a prior oxidative stress state was observed, by the elevated expression of 8-OHdG after 4 h of exposure. While this further supports the variability of effects in germ cells of different stages, we suggest that oxidative stress could be a likely mediator of damage in the male germ line.

The body of evidence revealing the genotoxic impacts of RF-EMR in spermatozoa and other cells is steadily growing (15–17, 32, 40, 54). However, the need to understand the physicobiological details of how non-ionizing radiation results in cellular damage remains unmet. Elucidating such a mechanism has been confounded by the considerable amount of conflicting data published to date (55). The difficulty in establishing a recognized mechanism is a major constraint when examining the potential clinical impacts of research in this field. This now not only warrants the investigation into potentially new safe exposure levels but also highlights the importance of probing the mechanisms of action.

### Origin of RF-EMR induced mitochondrial ROS production in male germ cells

To the best of our knowledge, the present study provides the first evidence that RF-EMR is capable of inducing mitochondrial ROS generation in precursor mouse germ cells (Figures 1A–C). The fact that similar responses were not elicited in any of the somatic cell lines examined (Figures 1D–F) suggests the male reproductive system may possess a unique vulnerability to RF-EMR and therefore supports the male germline as a potentially sensitive model system. Within the germ cell types examined, the spermatogonial-like GC1 cell line appeared more susceptible to RF-EMR than that of the later stage spermatocyte-like GC2 cells, as indicated by the earlier onset of elevated levels of mitochondrial ROS (2 vs. 4 h, respectively). One explanation for this may involve the morphological change of the germ cell mitochondria following progression to the spermatocyte stage, whereupon these organelles experience increased vacuolarization (56). Irrespective of this, the contrasting outcomes highlight that the effect of RF-EMR may vary greatly, depending on the stage of germ cell development experiencing RF-EMR exposure. On one hand, it has been well-established that isolated spermatozoa are susceptible to elevated ROS production (51, 57, 58) because of their lack of intrinsic antioxidant defense, but, *in vivo*, germ cells may be protected by the reproductive system through the provision of antioxidant protection, including superoxide dismutase and glutathione peroxidase (59).

It has previously been reported that spermatozoa exhibit a particular susceptibility to RF-EMR, revolving around mitochondrial dysfunction (13). The mouse model used in this study may exhibit some resistance compared to human spermatozoa, where these cells experienced no substantial increase in mitochondrial ROS generation following RF-EMR exposure in this study (Figure 5A). In line with the high variation in overall sperm cell quality in the human compared to the mouse, the lack of mitochondrial dysfunction in comparison in the mouse may reflect the relatively poor capacity of human sperm mitochondria to control the flow of electrons through the ETC (50). This in turn may reflect a heavier reliance of human spermatozoa on glycolysis for ATP production in comparison to murine spermatozoa (60). As a consequence of this pattern of metabolism, human sperm mitochondria may not only leak electrons more readily than their mouse counterparts but also be less competent at dealing with mitochondrial dysfunction induced by RF-EMR. Furthermore, it should be noted that for human spermatozoa (13) utilized extended exposure periods in comparison to this study (16 vs. 4 h) and higher intensities of exposure (up to 27.5 W/kg). This difference in exposure is likely to be the major reason for the discrete set of results generated between these two studies. However, this disparity is unable to be addressed as mouse spermatozoa do not survive long enough *in vitro* for such extended exposure times to be assessed.

Within the mitochondria, electron flow is generally strictly regulated. Any interruption of electron transport and associated electron leakage would be expected to elevate the production of ROS. To probe the origin of ROS under RF-EMR exposure we utilized inhibitors of Complex I, rotenone (Figures 2A,B), and Complex III, antimycin A (Figures 2C,D) in both cell lines. These compounds work to impede the flow of electrons through the ETC, by inhibiting the oxidation of the electron carrier ubiquinone in key intermediate sites of these complexes (61). As expected, we documented an induction of ROS in unexposed cells treated with rotenone, and to a lesser, yet still significant, extent with antimycin A. In this study, the ability of RF-EMR to induce additional ROS production in the presence of antimycin A within both GC1 and GC2 cell lines (Figures 2C,D) suggests that this ETC complex may be a key target for RF-EMR induced mitochondrial dysfunction. Furthermore, we document a different profile of ROS production in GC1 cells than in GC2 when treated with this combination of antimycin A and RF-EMR. Again, this is likely to do with their differences in mitochondrial architecture as these cells mature from spermatogonia to spermatocytes (56). While Complex I is responsible for a majority of the overt ROS leakage involved during normal mammalian cellular respiration, perturbation of Complex III fails to induce global oxidative stress in spermatozoa (50, 62–64). Furthermore, complete inhibition of Complex III alone does not induce downstream peroxidative damage to the sperm lipid membranes, but does encourage mitochondrial ROS production (50). This aligns with the data of the present study and may be accounted for by the leakage of electrons via this complex to the intermembrane space of the mitochondria, where they encounter the defenses of the mitochondrial antioxidant pool (50). Therefore, the increases in ROS levels observed following irradiation, in the presence of antimycin A, uncovers Complex III as a potential biological target for RF-EMR.

In order to add strength to this observation, we examined the impact of using succinate as an energy substrate for germ cell metabolism (Figures 2E,F). While both GC1 and GC2 cells displayed no significant increases in mitochondrial ROS generation when exposed to RF-EMR in the presence of succinate, a significant response was observed when such exposures were conducted in the presence of glucose. With succinate as the energy substrate, the majority of ROS generation has previously been attributed to Complex I (~90%), with a modest portion liberated at Complex III (~10%) (61). In the presence of glucose, where NADH is produced as the major electron source, the elevated mitochondrial ROS generation resulting from the exposure to RF-EMR is therefore likely to have been driven by Complex III, possibly involving the reduction of ubiquinone to ubiquinol (50, 61). This can be further rationalized by the absence of mitochondrial ROS production in both GC1 and GC2 cells following RF-EMR exposure when succinate is utilized as an energy source. Succinate metabolism drives electrons through Complex II and ROS production via this pathway has been characterized via the flow of electrons to Complex I via a mechanism of reverse electron flow (61, 65). In light of our observations, Complex I does not appear to be sensitive to RF-EMR exposure and provides further evidence that processes involving Complex III are responsible for the ROS associate with exposure to RF-EMR. The nucleophile scavenger penicillamine, provided an antioxidant effect in GC2 cells after 4 h of exposure (Figure 3C). We would expect that this effect of penicillamine is facilitated through protecting proteins in complex II from alkylation by lipid peroxidation products and hence reducing mitochondrial ROS leakage (53). This data does not discount the potential role of complex II, however, the modest antioxidant effect observed, perhaps provides additional support for the prominent role of complex III in germ cells, under RF-EMR exposure.

### The relationship between RF-EMR and DNA damage

While the RF-EMR levels used in this study were capable of inducing elevated mitochondrial ROS in the vulnerable spermatogonia and spermatocyte germ cell stages, this was not apparent in the mature spermatozoa (Figure 5A) and did not translate to downstream effects on lipid peroxidation in any cell type (Figures 6B–D). This lack of oxidative damage in germ cells is consistent with Complex III being the source of electron leakage following the exposure of male germ cells to RF-EMR (50). Furthermore, we did not detect an increase in the presence of 8-OHdG in either germ cell line exposed to RF-EMR (Figure 4C), which may suggest these cells were capable of attenuating ROS propagation with their antioxidant defenses. While no comet sensitive DNA insults were detected for GC2 spermatocytes in our study (Figure 4B), Liu et al. (17, 41) have previously implicated RF-EMR in the formation of DNA fragmentation and the formation of oxidative DNA lesions in equivalent GC2 cell lines. Although our exposure conditions encompassed the same radiation intensity as in the Liu study, our exposure duration was four-fold shorter (6 vs. 24 h). Such timing may account for differences between our observations, but also demonstrates the importance for precise experimental design in this field. In marked contrast, the response profile elicited within mature spermatozoa suggests a state of oxidative stress, with enhanced detection of the oxidative DNA lesion 8-OHdG (Figure 7C). It has been previously suggested that RF-EMR induced oxidative stress is also a driver of DNA damage in spermatozoa (13). While our MitoSOX red assay was not capable of differentiating the “active” ROS generation after exposure (Figure 5A), the elevated expression of 8-OHdG observed is consistent with findings in previous studies by De Iuliis et al. (13) and Zalata et al. (18), which identify that human spermatozoa exposed to RF-EMR suffer DNA fragmentation and oxidation. In the former human *in vitro* study, however, DNA damage occurred in association with increased mitochondrial ROS production, supporting oxidative stress as a causal factor in this setting (13).

### RF-EMR compromises sperm function

The inhibition of Complex I in the ETC in human spermatozoa, independent of RF-EMR exposure, results in a pronounced elevation of ROS and concomitant reduction to sperm motility; effects that are not readily apparent upon comparable inhibition of Complex III (50). Indeed, numerous studies have reinforced a causal link between oxidative stress and motility loss (1, 13, 18, 27, 38, 53). As we state above, while we did not observe elevated real-time ROS production or markers of lipid peroxidation in spermatozoa exposed to RF-EMR, we did detect increased 8-OHdG (a stable and reliable oxidative stress marker), suggesting indirectly, that oxidative stress may still have an important role in the causal effects observed, including those leading to sperm motility loss. There are two main mechanisms implicated in the regulation of sperm motility, where motility is elevated during capacitation with the onset of tyrosine phosphorylation signaling (66), or inhibited during membrane peroxidation in the event of oxidative stress. Meanwhile, impeding tyrosine phosphorylation events in spermatozoa also has a negative impact on sperm motility (66). We demonstrated that exposure to RF-EMR did not impact spontaneous protein tyrosine phosphorylation levels in exposed spermatozoa (Figures 8E,F), further implicating oxidative stress as a contributing factor in motility loss. Further, this result may highlight the effect of RF-EMR in accelerating the normal reduction of sperm cell quality over time.

With regard to the vulnerability of mouse spermatozoa to RF-EMR, direct comparison to published literature is challenging as former studies have largely focused (~92% of studies) on either rat or human models [1, 13, 18, 30, reviewed in (55)]. In a study utilizing *in vivo* exposed Swiss mice, RF-EMR did not influence sperm motility or vitality, but these cells did present with extensive DNA degradation within the mitochondrial genome (32). Meanwhile, rat and human spermatozoa appear to exhibit a greater vulnerability to RF-EMR; which diminishes sperm motility, viability and exacerbates ROS production in these cells [1, 27, 61, 62, (13)]. Here, we add to the small pool of data reporting the effects of RF-EMR on mouse spermatozoa. Our data proposes that Complex III of the ETC is a potential biological target of RF-EMR and provides impetus for the continuation of studies to further contribute toward our understanding of this mechanism. Many studies have shown no impact of RF-EMR on DNA integrity. In contrast, our study supports the capability of RF-EMR to induce genotoxic effects thus, complementing an alternative body of evidence that details a range of impacts elicited by this insult. Importantly, these effects have been recorded across a range of RF-EME intensities, thus encouraging further exploration of the impact of this form of non-ionizing radiation on biological systems.

## Author contributions

BH conducted the experiments and drafted manuscript. BN contributed to experimental design and manuscript preparation. BK provided equipment and contributed to experimental design. RA contributed to experimental design, interpretation of data, and manuscript preparation. GD contributed toward experimental design, interpretation of data, and manuscript preparation.

### Conflict of interest statement

The authors declare that the research was conducted in the absence of any commercial or financial relationships that could be construed as a potential conflict of interest.

## Supplementary material

Supplementary Figure 1 Examination of the effect of exposing male germ cells and spermatozoa to an elevated dose of RF-EMR. The studies reported in Figure 1 of this manuscript were replicated on **(A)** GC1 **(B)** GC2 cell lines and **(C)** spermatozoa exposed to an elevated intensity of RF-EMR (1.5 W/kg). Mitochondrial ROS generation in both germ cell populations and spermatozoa was subsequently assessed with the MSR probe. ^**^*p* < 0.01, ^*^*p* < 0.05 compared to unexposed controls. **(D)** Media temperature for the RF-EMR exposed treatments in the waveguide, and control counterparts over the exposure time course. **(E)** Representative comet images of GC1 cells, both control and RF-EMR exposed. **(F)** Blot of hexokinase showing the loading in each lane.

Supplementary Figure 2 Cell viability under RF-EMR exposure. Associated viability counts were performed for all cell types exposed to RF-EMR treatment. **(A)** GC1, **(B)** GC2, **(C)** spermatogonia, **(D)** HEK293, **(E)** COV434, and **(F)** McCoy cells exposed to 0.15 W/kg RF-EMR (top box). **(G)** GC1, **(H)** GC2, and **(I)** spermatozoa exposed to 1.5 W/kg RF-EMR (bottom box).
