# Supplementary figures and images for: Probing the Origins of 1,800 MHz Radio Frequency Electromagnetic Radiation Induced Damage in Mouse Immortalized Germ Cells and Spermatozoa in vitro
Source: Front Public Health. 2018 Sep 21;6:270. doi: 10.3389/fpubh.2018.00270 (PMC6160547; doi:10.3389/fpubh.2018.00270)

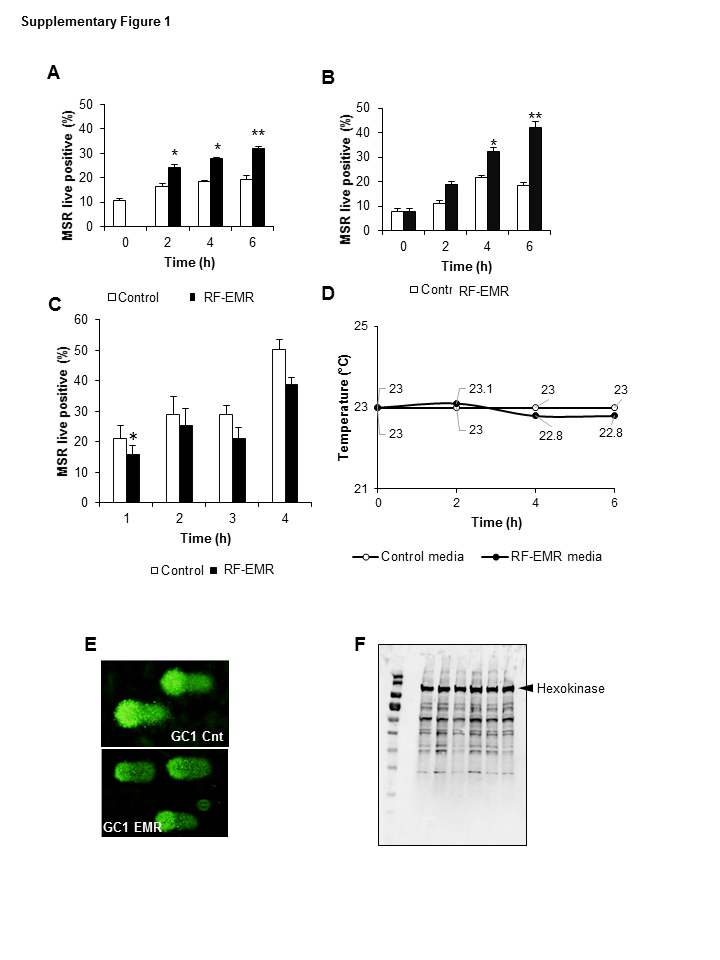

Supplement: Supplementary Figure 1 — Examination of the effect of exposing male germ cells and spermatozoa to an elevated dose of RF-EMR. The studies reported in Figure 1 of this manuscript were replicated on (A) GC1 (B) GC2 cell lines and (C) spermatozoa exposed to an elevated intensity of RF-EMR (1.5 W/kg). Mitochondrial ROS generation in both germ cell populations and spermatozoa was subsequently assessed with the MSR probe. **p < 0.01, *p < 0.05 compared to unexposed controls. (D) Media temperature for the RF-EMR exposed treatments in the waveguide, and control counterparts over the exposure time course. (E) Representative comet images of GC1 cells, both control and RF-EMR exposed. (F) Blot of hexokinase showing the loading in each lane. [file Image_1.TIF]

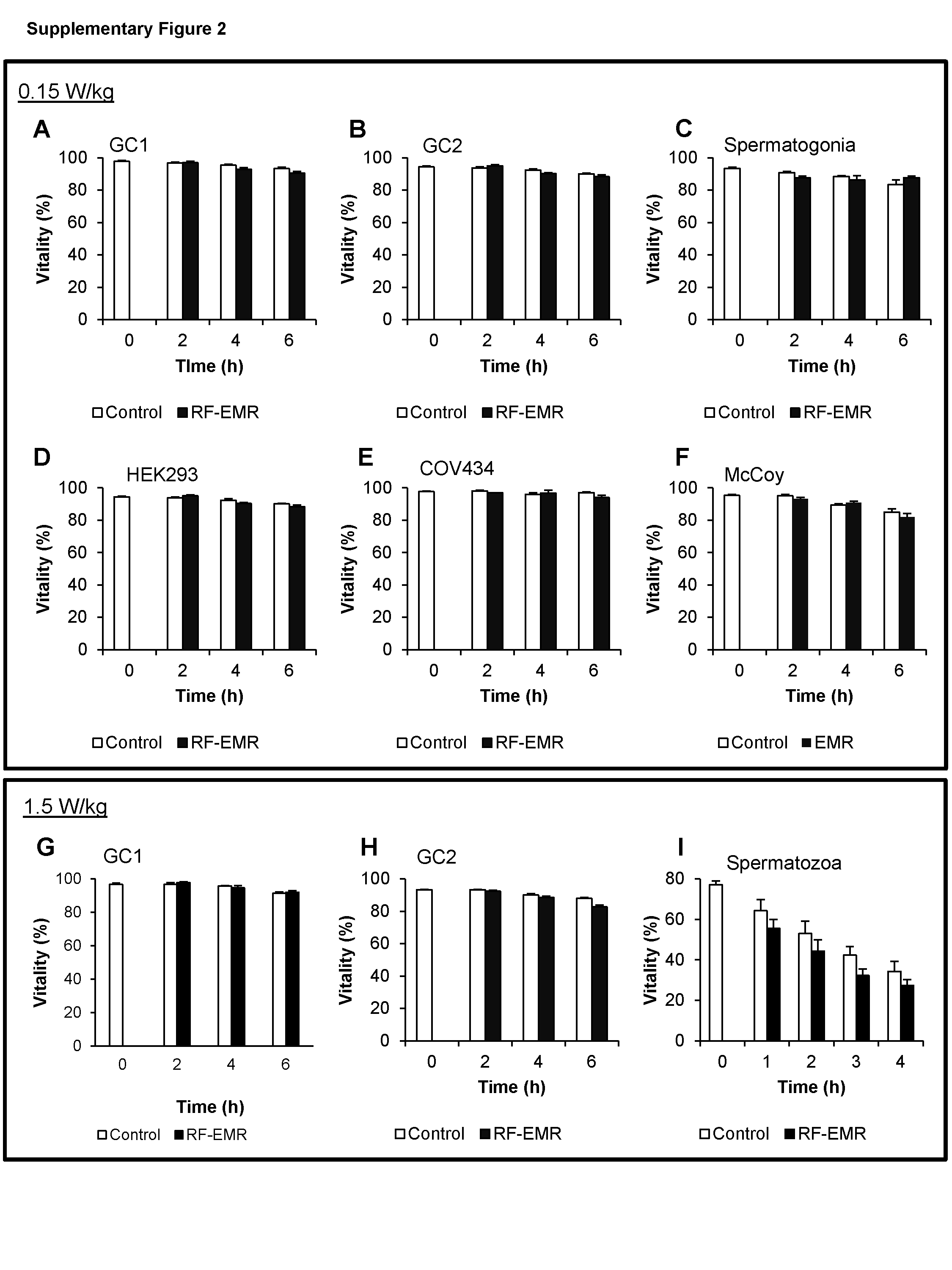

Supplement: Supplementary Figure 2 — Cell viability under RF-EMR exposure. Associated viability counts were performed for all cell types exposed to RF-EMR treatment. (A) GC1, (B) GC2, (C) spermatogonia, (D) HEK293, (E) COV434, and (F) McCoy cells exposed to 0.15 W/kg RF-EMR (top box). (G) GC1, (H) GC2, and (I) spermatozoa exposed to 1.5 W/kg RF-EMR (bottom box). [file Image_2.TIFF]
